# IL-33/ST2 Axis: A Potential Therapeutic Target in Neurodegenerative Diseases

**DOI:** 10.3390/biom13101494

**Published:** 2023-10-08

**Authors:** Zexi Jia, Mengtian Guo, Xintong Ge, Fanglian Chen, Ping Lei

**Affiliations:** 1 Department of Geriatrics, Tianjin Medical University General Hospital, Tianjin 300052, China; jiazexi1999@tmu.edu.cn (Z.J.); xge@tmu.edu.cn (X.G.); 2 Tianjin Geriatrics Institute, Tianjin Medical University General Hospital, Tianjin 300052, China; 3 Department of Internal Medicine, Beijing Chao-Yang Hospital, Capital Medical University, Beijing 100054, China; guomengtian1003@163.com; 4 Tianjin Neurological Institute, Tianjin 300052, China

**Keywords:** IL-33, ST2, neurodegenerative diseases, immune, therapeutic target

## Abstract

Interleukin 33 (IL-33) belongs to the IL-1 family and is localized in the nucleus. IL-33 is primarily composed of three distinct domains, namely the N-terminal domain responsible for nuclear localization, the intermediate sense protease domain, and the C-terminal cytokine domain. Its specific receptor is the suppression of tumorigenicity 2 (ST2), which is detected in serum-stimulated fibroblasts and oncogenes. While most other cytokines are actively produced in cells, IL-33 is passively produced in response to tissue damage or cell necrosis, thereby suggesting its role as an alarm following cell infection, stress, or trauma. IL-33 plays a crucial role in congenital and acquired immunity, which assists in the response to environmental stress and maintains tissue homeostasis. IL-33/ST2 interaction further produces many pro-inflammatory cytokines. Moreover, IL-33 is crucial for central nervous system (CNS) homeostasis and the pathogenic mechanisms underlying CNS degenerative disorders. The present work summarizes the structure of IL-33, its fundamental activities, and its role in immunoregulation and neurodegenerative diseases. Therefore, this work proposes that IL-33 may play a role in the pathogenic mechanism of diseases and can be used in the development of treatment strategies.

## 1. Introduction

Interleukin 33 (IL-33) is a member of the IL-1 family and is produced in diverse tissues such as the nervous system [1,2]. It is an alarmin cytokine that promotes inflammatory responses by warning the immune system after trauma to the endothelium, epithelium, fibroblasts, adipocytes, smooth muscle cells, etc. [3,4,5]. IL-33 was first identified as a clone *DVS27* in the vasospasm cerebral artery of a canine subarachnoid hemorrhage (SAH) model in 1999 [6]. In 2005, the mining of public genome databases by using β-trefoil fold protein sequences derived from the structural overlay of IL-1 and fibroblast growth factor (FGF) proteins led to the discovery of a new IL-1 family member in a canine-derived cDNA library [7]. Sequence analysis has demonstrated that the protein encompasses a 30 kDa full-length pre-domain which is the precursor to IL-33. Additionally, it has been found that IL-33 has the capability to function as a ligand for ST2L (transmembrane ST2, a membrane-bound receptor) [8]. The IL-33-encoding gene is composed of eight exons in chromosome 9 short arm, at 9p41.1 [9]. It possesses one N-terminal domain with an 18-kDa C-terminal region with abundant β sheets and one chromatin-binding motif [10]. In humans, IL33 mRNA encodes a 270-residue protein, whereas in mice, it encodes 266 residues [11]. IL-33 exhibits biological activity and is regulated by a C-terminal IL-1-like domain [12]. IL-33 exhibits nuclear localization and acts as a transcriptional repressor [8,13]. IL-33 is a chromatin-binding transcriptional regulator highly expressed in the nuclei of endothelial cells. For example, when tissue damage or inflammation occurs, nuclear IL-33 becomes a transcription factor of NF-kB p65 and induces endothelial cell activation [14]. In its role as a transcription factor, it mediates cell signal transduction by releasing molecules that initiate signaling in receptor cells, thereby enabling cell-to-cell communication. Target cells generally contain ST2 receptors, such as Th2 cells, mast cells (MCs), Th1 cells, Treg cells, ILC2s, CD8^+^ T cells, and NK cells. Additionally, it plays a key role in regulating the cell cycle, particularly in the context of oncogenes or tumor suppressor genes. Moreover, it can modulate gene expression in host cells and facilitate pathogenesis. Furthermore, it can also participate in the downstream signaling cascade of environmental stimuli [15]. In an in vivo study, IL-33 translation and caspase-1 administration produced an 18-kD processed protein that activated ST2 receptor 1. IL-33 can trigger the expression of IL-4, IL-5, and IL-13, which can result in significant physiological and pathological alterations in mucosal organogenesis [8]. Phylogenetic analysis has demonstrated that the IL-33 protein is evolutionarily conserved among mammals [12]. Moreover, the IL-33 structure is closely related to those of IL-1b and IL-18 because IL-33 is a prodomain-containing protein that is processed for optimal biological activity [8]. In both human and mouse tissues, IL-33 is constitutively expressed at high levels in the nuclei of different cell types in the steady-state [9], such as epithelial cells derived from bronchi or bronchioles, smooth muscle cells (SMCs), and fibroblasts [8]. IL-33 secreted by astrocytes maintains network homeostasis by regulating the structural and functional changes in hippocampal synapses [16].

ST2, also known as IL-1 receptor-like-1 (IL-1RL1) and T1 [17], is produced by different immunocytes, such as T cells [18], macrophages [19], and mast cells [20]. As an IL-1R family member, ST2 also belongs to the Toll-like receptor (TLR) superfamily. The ST2 gene was first discovered in mouse fibroblasts as a serum-inducible secretory protein that was spliced to form a soluble functional fraction (sST2), membrane-bound receptor (ST2L), or the variant ST2 (ST2V) [21,22]. ST2 is present in two splicing variants, namely sST2, which functions as a decoy receptor and sequesters free IL-33 without transmitting signaling, and ST2L, which activates the MyD88/NF- κ B signaling pathway, thus enhancing the functionality of mast cells, Th2 cells, Tregs, and type 2 innate lymphoid cells [23]. The ability of ST2 to bind IL-33 indicates that this receptor is part of a functional IL-33 receptor complex [8,24]. IL-33 forms heterodimers with the corresponding receptor complex ST2/(IL-1RAP), which conducts activation signals into the cells. This results in the dimerization of the Toll/interleukin-1 receptor (TIR), thereby recruiting bone marrow differentiation primary response protein 88 (MyD88) and activating IL-1R-related kinases. Finally, mitogen-activated protein kinases (MAPKs) are activated to exert their corresponding biological effects [2].

IL-33 is related to pathogenic mechanisms in neurodegenerative disorders such as Parkinson’s disease (PD), Alzheimer’s disease (AD), multiple sclerosis (MS), Huntington’s disease (HD), chronic traumatic encephalopathy (CTE), and amyotrophic lateral sclerosis (ALS). IL-33 also plays a dual role in neuroprotection and neurotoxicity in neurodegenerative diseases [25]. The present work summarizes the existing knowledge regarding the IL-33/ST2 pathway in neurodegenerative disorders and the potential of IL-33 in treating such disorders.

## 2. IL-33 Distribution in Human and Mice Tissues and Cells

The IL-33 protein is mainly localized in the cell nuclei of human and mouse cells [26,27] (see Table 1). It is synthesized in perivascular cells, stromal fibroblasts, adipocytes, epithelial cells, cancer cells, and endothelial cells [28,29,30]. Under normal conditions, IL-33 expression is detected in mouse alveolar type II epithelial cells [31]; IL-33 protein is abundant in cardiac fibroblasts. The gene expression of IL-33 is higher in cardiac fibroblasts than in cardiomyocytes. IL-33 is associated with chromatin and is abundantly expressed in the vascular endothelial cells (VECs) of most healthy human samples. Apart from endothelial cells, IL-33 is detected in the fibroblasts of lymphoid tissues and in the epithelial cells of salivary glands, stomach, and tonsils [32]. Endogenous IL-33 is highly expressed in mouse epithelial barrier tissues, lymphoid organs, brain, embryos, and inflamed tissues [28]. IL-33 has two subtypes, IL-33a and IL-33b, which are regulated by distinct promoters. Neurons predominantly express IL-33b, while astrocytes produce IL-33a. Furthermore, in one study, immunostaining revealed that IL-33 expression is most abundant in the dentate gyrus region [33]. IL-33 is predominantly expressed in astrocytes and oligodendrocytes in the brain but is also expressed in oligo, microglia, and neurons at lower levels [2,34]. Upon injury to the central nervous system, IL-33 is rapidly released, acting on astrocytes and microglia. This subsequently induces the production of chemokines that are essential for monocyte recruitment.

## 3. IL-33 Signaling Pathway

**Figure 1:**
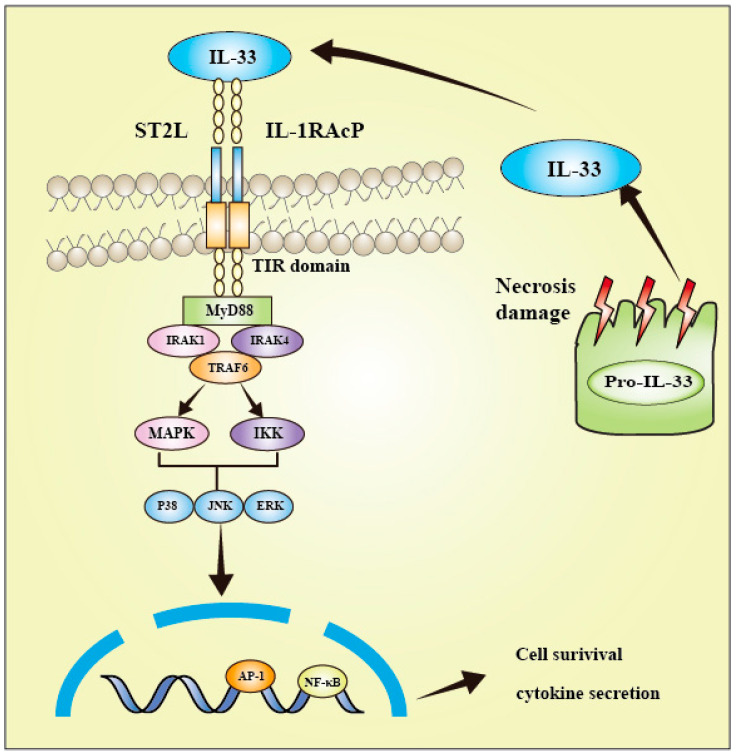
Interleukin-33 signaling pathway. The colocalization of ST2 and IL-1RAcP clusters their TIR domains, thereby facilitating the recruitment of MyD88, IRAK1, IRAK4, and TRAF6. Subsequently, several signal transduction proteins, such as downstream NF-κB, JNK, p38, and ERK, are activated, leading to inflammation.

IL-33 is a member of the IL-1 family and has emerged as a crucial promoter in multiple inflammatory and immune-mediated pathological scenarios. The widespread expression of ST2 receptors is advantageous for IL-33 to engage in the pathogenesis of diverse diseases. The IL-33/ST2L axis has been extensively investigated, and it has been implicated in the pathogenesis of several illnesses, as well as in the development, invasion, and metastasis of various cancers. The IL-33 signaling pathway is different from that of Th2 cytokines (Figure 1). IL-33 binds to the transmembrane receptor complex consisting of ST2 and IL-1RAcP. This leads to the dimerization of the TIR domain of both ST2 and IL-1RAcP receptors, resulting in the recruitment of MyD88, TNF receptor-associated factor 6 (TRAF6), IL-1 receptor-associated kinase 1 (IRAK1), and IRAK4 [26]. As a consequence, the downstream nuclear factor-κB (NF-κB) and MAPK pathways are activated. Such pathways jointly modulate cell proliferation, differentiation, immunity, and stress [9,36,37]. Moreover, they modulate numerous pathophysiological actions and physiological effects, such as apoptosis, necrosis, inflammation, cancer cell migration, invasion, and tumorigenesis. For instance, IL-33 is released upon cell necrosis, and the precursor form is enzymatically processed and then drives inflammation as a damage-associated molecular pattern. A large amount of IL-33 is released outside the cell, causing multiple immune responses during cell damage [27].

## 4. IL-33 and Immune Cells

**Figure 2:**
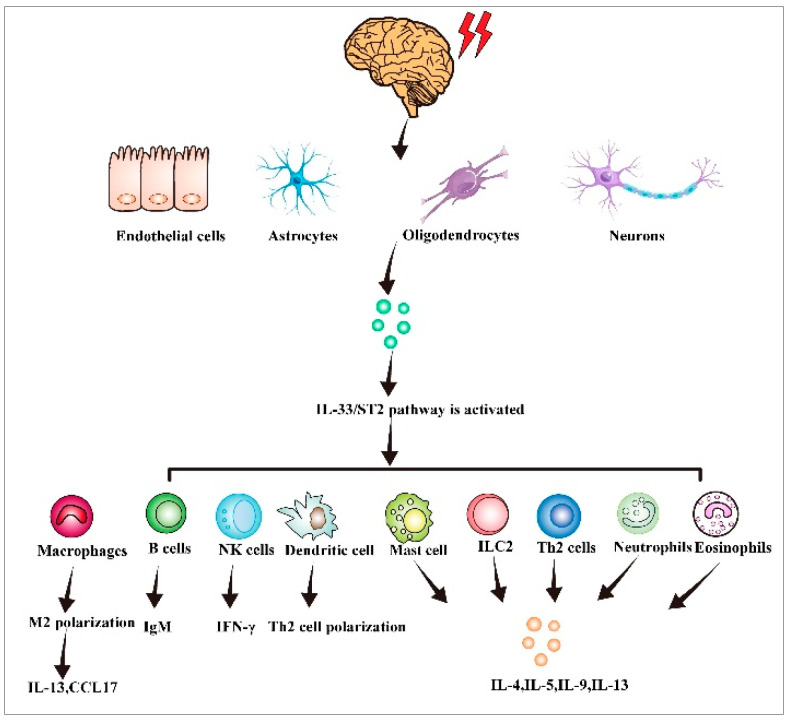
IL-33 activates immune cells and triggers an immune response. When tissues and cells are damaged or stimulated by the outside world, IL-33 is produced in endothelial fibroblasts, astrocytes, oligodendrocytes, and neurons. The IL-33/ST2 axis is activated, thereby stimulating B cells, NK cells, dendritic cells, type 2 innate lymphocytes, neutrophils, and eosinophils, leading to an immune inflammatory response.

An increasing body of evidence suggests that IL-33 plays a significant role in the development of immune responses. IL-33 is a pleiotropic cytokine that links different immunocytes of the innate and adaptive immune system (Figure 2) [38]. IL-33 activates many immunocytes, including ILC2s, Th2 cells, eosinophils, basophils, dendritic cell subsets, mast cells, and myeloid-derived suppressor cells [39]. When ILC2 is activated by IL-33, it releases a variety of cytokines, such as IL-5, IL-6, and IL-13 [40]. Regulatory T cells (Tregs) mainly inhibit the response of T cells to themselves, symbiotic microbiota, and dietary and environmental antigens. Treg cells also promote repair under inflammatory conditions and organ damage [41]. IL-33 promotes epidermal growth factor-like molecule amphiregulin (AREG) growth, thereby promoting immunoregulatory function and tissue repair [42]. Viral infection triggers IL-33 production in cells. IL-33 promotes primary effector amplification and CTL activation through its specific receptor ST2, thereby enhancing effector differentiation and antiviral cytokine production [43]. The synergistic effect of IL-33 and IL-12 enhances CD8^+^T cell effector activity and IFN-γ production [44]. Basophils release chemokines such as heparin, histamine, and leukotriene, thereby causing hypersensitive reactions such as asthma. After activation, IL-33 induces basophils to stimulate IL-1 receptors and Th2-type cytokine expression at the mRNA and protein levels [45]. We administered IL-33 into mice by using various methods and observed massive eosinophil infiltration in tissue, increased typical type 2 cytokine levels, and epithelial goblet hyperplasia. IL-33 effectively induced CD11b expression and eosinophil adhesion while enhancing eosinophil survival. Moreover, the IL-33-ST2 pathway may regulate eosinophil biology during the pathogenic mechanism of Th2-biased allergic disorders [46]. IL-33 treatment induced OX40L expression in specific organs together with Tregs and Th2 cell expansion on ILC2 cells [47]. Mast cells are a type of innate immune and inflammatory cells that have the ability to monitor the immune system. Proteases are immune-regulating molecules that are pre-formed in the cytoplasmic granules of mast cells. They are stored in the degranulation of mast cells in an active form and release extracellular substances when degranulation occurs, which serves as a key sign of mast cell degranulation [48,49,50]. Mast cells can be extensively expressed on microvessels in visceral submucosa and skin. Mast cells produce various cytokines, are related to immunomodulation, and express several IgE Fc receptors to release allergic mediators. IL-33 receptor and the related protein ST2 directly activate mast cells and prompt their responses to additional proinflammatory signals; both are upregulated in MCs. One study suggested that the IL-33/ST2 axis promotes mast cell survival [20]. Moreover, the p38-MK2/3 signaling module plays a crucial role in mediating IL-33-mediated cytokine generation in dendritic cells (DCs). MK2/3 inhibition is a promising treatment measure for IL-33-driven disease [51]. In one particular study, IL-33 promoted cytokine generation in ILC2 and activated CD11b^+^ DCs [52].

## 5. Role of IL-33 in Neurodegenerative Disorders

Neurodegenerative diseases are mostly sporadic and genetic disorders of the central nervous system that typically manifest as a gradual and progressive decline in the function of particular neuronal populations and their connections. These diseases share common pathological traits, such as mitochondrial dysfunction, oxidative stress (OS), and inflammatory responses to excitatory toxins. Examples of neurodegenerative diseases include AD, PD, MS, ALS, and HD (see Table 2). In the past decade, many studies have focused on the pathogenesis of neurodegenerative diseases, revealing the eight hallmarks of ND: pathological protein aggregation, synaptic and neural network dysfunction, adverse proteostasis, cycloskeleton abnormalities, altered energy homeostasis, DNA and RNA defects, inflammation, and neural cell death. In addition, the necessity of multi-targeted and personalized treatment was proposed, providing new ideas for future clinical translation [53]. Although IL-33 is highly expressed in CNS [8,35], its role in CNS degeneration remains unexplained. The robust expression of IL-33 in the brain and spinal cord is a key molecular signal that coordinates the interaction between the immune system and the central nervous system. The mechanism underlying the IL-33/ST2 axis in the central nervous system is complex and involves a multicellular network consisting of neurons, oligodendrocytes, astrocytes, and microglia. The IL-33/ST2 signaling pathway is involved in regulating excitatory sensory neurons, axonal myelin formation, and synaptic homeostasis. The disruption of this signaling pathway may result in central nervous system and behavioral disorders [35]. The cellular mechanism by which IL-33 promotes stable synaptic remodeling via microglia demonstrates that neuron-derived IL-33 stimulates the extracellular matrix of microglial phagocytes, thereby promoting increased synaptic plasticity [33]. The expression level of IL-33 may vary depending on the disease, cell type or sample source. Specifically, the expression level of IL-33 may vary from the different pathogeneses in the different subtypes of the various neurodegenerative diseases. However, there are also some obvious commonalities between the different NDs. For example, glial cell maturation factor (GMF) is upregulated in CNS, and IL-33 augments GMF-mediated neuroinflammation in ND [54]. GMF may enter the brain by activating glial cells, neurons, mast cells, and T cells to induce or enhance neuroinflammation. The GMF-dependent expression and secretion of inflammatory cytokines/chemokines, such as TNF-α, IL-1β, and IL-6, is cytotoxic to oligodendrocytes, the myelin-forming cells, and neurons [55,56]. IL-33 is a key cytokine that coordinates the exchange between the immune and central nervous system. IL-33 has also been shown to have direct effects on CNS glia, as IL-33 induced various innate immune effectors in CNS glia, according to the authors of [57]. The precise pathophysiological activity of the IL-33/ST2 signaling pathway in neurodegenerative diseases should be further explored, and IL-33 could be a promising prognostic marker and therapeutic target for neurodegenerative disorders [34].

### 5.1. AD

**Figure 3:**
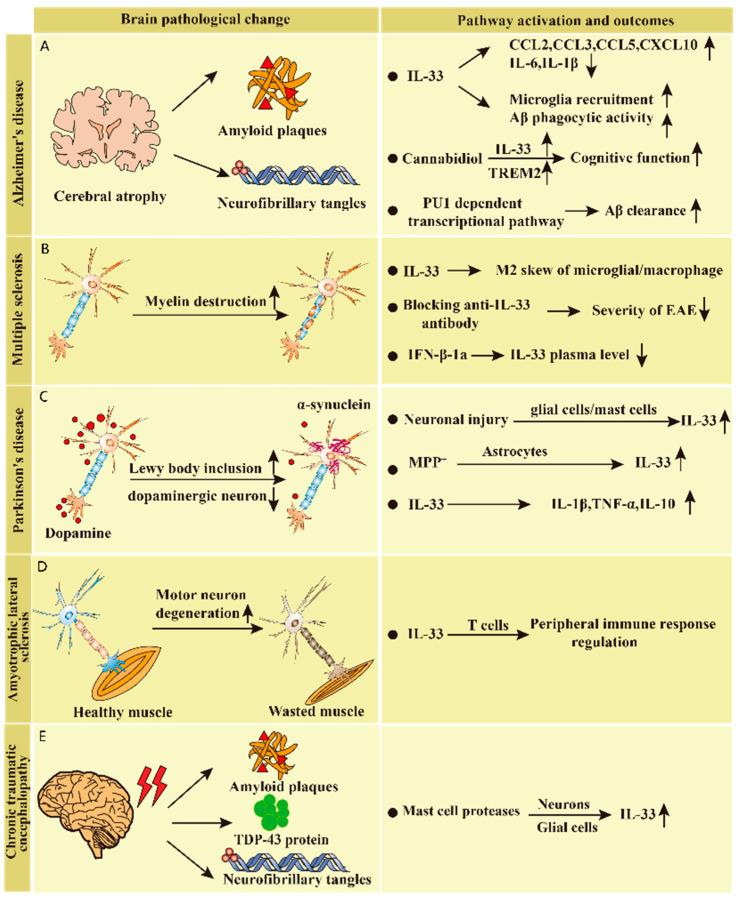
A schematic overview of the role of IL-33 in neurodegenerative disease and potential signaling mechanisms. The pathological changes, pathway activation, and outcomes of each kind of neurodegenerative disease are displayed. (**A**–**E**) show the role of IL-33 in different neurodegenerative diseases.

AD is the most common cause of dementia and affects millions of people worldwide [75]. According to the World Health Organization, the global incidence of AD is approximately 4–7% among people aged >65 years. The incidence of AD increases with age; in people aged over 85 years, AD incidence ranges from 20% to 30% [76,77,78]. If medical breakthroughs to prevent, slow, or cure AD are not achieved, the number of AD patients could reach 13.8 million by the middle of the century [79]. The typical pathological features of AD include atrophy of the temporal, parietal, and prefrontal lobes, age-related plaques, neurofibrillary tangles, decreased neurons, granular vacuolar degeneration, and vascular amyloid changes. The chronic increase in proinflammatory mediators induces neurotoxic Aβ and plaque formation in AD. The most common clinical manifestations of AD include cognitive dysfunction (mainly in the form of memory loss), orientation impairment, and impaired calculation ability. IL-33 exhibits a constitutive expression in the CNS and is a key factor mediating neuropathological injury response in glial cells. Under normal conditions, IL-33 is constitutively expressed by neurons, microglia, astrocytes, and oligodendrocytes, while ST2 is expressed by microglia, neurons astrocytes and oligodendrocytes. However, the IL-33 level is correlated with cognitive protection among AD cases [80,81]. The expression of IL-33 mRNA and protein is significantly increased in CNS glia and astrocytes following exposure to pathogen-associated molecular patterns (PAMPs) in AD. Notably, IL-33 is an alarmin cytokine that induces inflammatory molecule release from glial cells. In patients with AD, significant changes in the levels of IL-1 family cytokines and receptors in circulation have been observed, indicating their use as a marker to assess AD progression [58]. In glial cells, IL-33 is involved in the autocrine and/or paracrine pathway, which facilitates cerebral neuroinflammation among AD cases [69,82]. A previous research study indicated that IL-33, which was expressed by 75% astrocytes in the aged brains, played a crucial role in the process of aged neuron repair. Mice lacking IL-33 develop AD-like disease after 60–80 weeks, with the characteristics of tau abnormalities and massive neuronal loss in the hippocampus and cerebral cortex, cognitive or memory impairment, and an increased rate of aging. Neurons in IL-33-deficient mice rapidly accumulate abnormal tau, numerous DNA double-strand breaks, and abnormal autophagic vesicles [60]. IL-33 can enable beta-amyloid (A β) protein decreases and can activate the phagocytosis of microglia, thus playing a neuroprotective role [61]. In an animal model of AD, the cytokine IL-33 has been shown to promote the microglial phagocytosis of beta-amyloid and reduce their pro-inflammatory response [83]. IL-33 polarizes microglia or macrophages to the anti-inflammatory phenotype and reduces the levels of proinflammatory factors (such as IL-1β and IL-6). An enhancement in the phagocytic activity of microglia can improve clinical symptoms and reduce soluble Aβ levels and plaque formation. In AD, impaired microglial clearance activity leads to the accumulation of Aβ, and astrocytes may regulate microglial phagocytosis by releasing molecules such as IL-33. Therefore, IL-33 upregulation is a promising strategy for reducing neuroinflammation. Several studies have demonstrated that IL-33 can induce microglial subsets with enhanced phagocytic activity via reprogramming their epigenetic and transcriptomic profiles, thereby reducing the pathological burden of AD. In addition, the Major Histocompatibility Complex (MHC) class II also plays an important role in AD. This is a specific type of molecular complex consisting of MHC class II molecules and antigenic peptides present on the surface of antigen-presenting cells. MHC class II molecules are widely expressed on the surface of antigen-presenting cells and are critical for antigen processing and presentation. PU.1 is a transcription factor that is specific to hematopoietic cells and belongs to the conserved DNA-binding protein Ets family [84]. IL-33 reshapes the PU.1 binding landscape and chromatin in microglia, thereby inducing IL-33-reactive microglia with the upregulation of homeostasis signature and histocompatibility complex class II genes, together with increased Aβ phagocytosis and clearance [85]. Pu.1-dependent transcriptional pathways in AD promote IL-33-mediated functional status transitions in microglial cells, thereby improving phagocytic activity and enhancing Aβ clearance [64]. Furthermore, IL-33 molecules act as a control agent and accelerate abnormal protein excretion from the brain; however, the increase in brain waste is inversely proportional to the decrease in IL-33 levels. This may include injecting IL-33 into the blood–brain barrier (BBB) to increase its circulation in the lymphatic system and to evaluate the efficiency of plaque protein excretion from the brain [86]. Previous studies have indicated that the proteins TREM2 and IL-33 play a critical role in the ability of immune cells in the brain to phagocytose dead cells and other debris. Furthermore, the levels of both proteins are decreased in AD patients. Cnnabidiol (CBD) treatment enhances TREM2 and IL-33 expression, alleviates neurocognitive functional impairment by regulating glial cell function, improves AD symptoms, and inhibits the expression of pro-inflammatory factor (IL-6) in white blood cells (WBCs) in peripheral blood; therefore, CBD treatment can be used in the clinical settings [62,87]. In conclusion, IL-33 plays an essential role in AD and can be a promising therapeutic target in clinical translation (Figure 3A).

### 5.2. MS

MS is a chronic autoimmune disorder characterized by inflammation, demyelination, and glial scarring in multiple areas of the central nervous system’s white matter [88]. MS affects people’s daily lives by causing complications such as neurological disorders, abnormal sweating, orthostatic dysbiosis, gastrointestinal symptoms, and urinary and sexual dysfunction [89]. Although several non-pharmacological and pharmacological treatments effectively alleviate MS-related neurological symptoms, those associated with pathological mechanisms remain unexplored; moreover, etiological treatments and evaluation methods for the neurological function of patients with MS in clinical practice are lacking [88,90]. In patients with multiple sclerosis (MS), the expression of ST2 and IL-33 is upregulated in brain lesions compared to healthy individuals. However, their levels decrease after treatment with interferon β-1a, a polyethylene glycol interferon with potent immune-modulating effects that is used to treat recurrent MS in adults. Treatment with interferon β-1a has been shown to significantly reduce the recurrence rate of MS and slow down the progression of disability [91,92]. There are studies indicating that interferon β-1a treatment reduced the expression of IL-33 in the plasma and peripheral blood monocytes of MS patients. IL-33 may have a preventive effect on EAE during the initial (induction) stage of the disease. However, the presence of some inflammatory cells in the central nervous system may promote the development of the disease. Regarding MS disease patterns, a larger sample size may be required for effective statistical analysis [66,92]. The administration of IL-33 helps myelin repair by inducing the transcription of myelin genes and promoting the differentiation of OPC to form mature myelin forming cells, indicating its neuroprotective effect in MS [93]. NF-κB, a transcription factor mediating IL-33 transcription, exhibits upregulation in WBCs obtained from patients with MS. IL-33 expression is elevated in both normal-appearing white matter and plaque areas of MS, with astrocytes being the primary source of IL-33 expression in the central nervous system [66]. In a mouse model of MS, mast cells secrete cytokines such as IL-33, which in turn stimulate ST2, IL-2, and other cells to release molecules such as IL-4 and IL-13. IL-4 and IL-13 can inhibit Th17 cells, which are responsible for promoting the myelin sheath. Experimental autoimmune encephalomyelitis (EAE) is the most common animal model for MS. ST2 and IL-33 levels in the spinal cord in EAE mice were significantly increased in [94]. Apigenin and luteolin inhibit IL-33 expression through the suppression of IL-33 mRNA and protein levels in microglial cells, thereby inhibiting IL-33 secretion by microglia [95]. In experimental models of CNS injury, IL-33 has been shown to promote neural repair, and we hypothesize that compounds that induce IL-33 may also enhance myelin regeneration. Anacardic acid has been identified as a candidate molecule that can induce the production of IL-33. When added to cultured oligodendrocyte precursor somatic cells (OPCs), Anacardic acid rapidly increases the expression of genes and proteins involved in myelin formation, suggesting that it directly induces myelin gene expression [96]. In mouse splenic tissues and lymph nodes treated with IL-33, the inhibition of IFN-γ and IL-17 expression polarized macrophages into the M2 phenotype and attenuated EAE development, demonstrating its role in autoimmune CNS diseases [67]. A recent study suggested that changed IL-33 subcellular localization alone may affect immune homeostasis [97]. Therefore, elevated IL-33/ST2 mRNA levels were observed in the spinal cord of EAE rats compared with healthy controls; however, the levels remarkably decreased among matrine (MAT)-treated rats. MAT is a natural compound (a quinoline alkaloid extracted from Radix Sophorae Flave). These results indicate the role of MAT exposure in modulating the IL-33/ST2 pathway [68]. The role of IL-33 in initial demyelination or subsequent in vivo neurodegeneration remains unknown and should be further investigated (Figure 3B).

### 5.3. PD

PD or paralysis agitans is a neurodegenerative disease characterized by CNS dopaminergic neuron loss, resulting in quiescent tremors, bradykinesia, myotonia, motion initiation difficulty, and various other motor and nonmotor symptoms [80]. PD exhibits inflammation-mediated dopaminergic neurodegeneration in substantia nigra and intracerebral Lewy bodies [81]. PD is characterized by the progressive loss of intracellular protein-like inclusions and dopaminergic neurons, and the etiology of the disease may be genetic or environmental [58]. In PD patients, the concentration of IL-33 in serum is significantly increased compared to healthy individuals, and the expression of IL-33 is upregulated in the midbrain and striatum of PD patients’ brains. Neuronal injury and neurodegeneration may also contribute to the release of IL-33 from glial cells and mast cells in the brain [59]. IL-33 induces microglia growth and promotes the expression of pro-inflammatory factors IL-1β and TNF-α, along with anti-inflammatory factor IL-10 [69]. Nonetheless, the protective or disease-enhancing role of IL-33 in PD development remains unclear. In PD, in vitro studies have shown that MPP^+^, a metabolite of the parkinsonian neurotoxin 1-methyl-4-phenyl-1,2,3,6 tetrahydropyridine (MPTP), can induce astrocytes to release IL-33 [98]. Mast cells, a population of cells that express receptors for IL-33 and are activated in PD brains, may contribute to neuroinflammation during PD development. The mouse mast cell proteolytic enzymes MMCP-6 and MMCP-7 can activate mouse glia and astrocytes, which in turn release IL-33. The selective activation of IL-33 in mast cells induces astrocyte activation and p38 and NF-κB upregulation; these are crucial signaling mechanisms for pro-inflammatory cytokines. The pleiotropic Il-33 activates other types of nerve cells, such as neurons or microglia [48]. Mast cells are activated by the dopaminergic Toxin 1-Methyl-4-Phenylpyridiniumd, glial maturation factor, and α-Synuclein; these produce inflammatory factors during PD pathogenesis [98]. IL-33 enhances neuroinflammation mediated by GMF [60]. Neurons and glial cells exhibit interactions with mass cells, which promote neuroinflammation progression and are the novel targets of PD treatment [83] (Figure 3C).

### 5.4. ALS

ALS or Lou Gehrig’s disease is a progressive, adult-onset degenerative disorder that causes mobility problems, respiratory failure, and, eventually, death [99]. The pathogenesis of ALS is related to genetic, cellular, and molecular mechanisms; however, its pathogenesis and pathological progression remain unclear [100]. Currently, genetic abnormalities in neuronal proteins are gaining researchers’ interest. The disease is characterized by motor neuron degeneration in the spinal cord and brain, leading to myasthenia. ALS targets the lower and upper motor neurons, thereby inducing muscle paralysis [101]. People survive for a median of 3–5 years after the symptom onset, with a lack of effective treatment [102]. One study suggested that the ALS progression rate is possibly associated with neuronal degeneration response, and acute phase reactants and cytokines levels in the blood may change its progression [103]. In another study, compared with healthy individuals, IL-33 expression significantly decreased in ALS cases because cell death was caused by apoptosis rather than necrosis [70]. IL-33 is subject to degradation by caspases. Recent research has revealed elevated levels of caspase 9 in the serum of individuals with ALS, suggesting that this may contribute to the degradation of IL-33 in these patients. IL-33 destruction is caused by caspase, whereas sST2 expression is remarkably elevated [72]. IL-33 regulates peripheral inflammatory responses, and it may not act directly on neurons or astrocytes but on peripheral T cells [71]. In the early stages of ALS, changes occur in the central nervous system barrier, including reduced tight junction proteins and abnormal ultrastructure, leading to increased permeability. The interaction between glial cells and peripheral immune cells in the CNS exacerbates inflammatory reactions, causing synaptic damage to motor neurons, neuromuscular junction dysfunction, and even motor neuron death [102,103]. T lymphocytes expressing CD6 receptors bind to endothelial cells expressing white cell adhesion molecules, enabling their entry into the brain parenchyma. CD8^+^ effector T cells recognize the self-peptide MHC-I complex on motor neurons and can infiltrate the CNS to cause motor neuron damage or death [104,105]. In a transgenic mouse model of ALS, treatment with IL-33 delayed disease onset in female mice and reduced the proportion of CD4^+^ and CD8^+^ T cell populations in the spleen and lymph nodes. The delayed disease onset was not due to the direct binding of IL-33 on CNS cells. Instead, a conditioned medium derived from IL-33-treated T cells decreased the expression of MCP-1, a chemokine that increases blood-CNS barrier permeability, indicating that IL-33 exerts its effects through peripheral T cell-mediated cytokines [71]. This suggests that strategies targeting peripheral immunity may be able to treat ALS (Figure 3D).

### 5.5. Other Types of Neurodegenerative Diseases

CTE has emerged in recent years. CTE exhibits hyperphosphorylated tau in NFTs, perivascular neurons, cellular processes, and astrocytes [106]. However, the incidence of CTE remains unclear, as the clinical diagnostic criteria is yet to be concluded. CTE occurs after repeated damage to the brain caused by frequent hits to the head and minor concussions. It usually occurs in athletes such as boxers and football players [107]. After CTE onset, mast cell proteases induce neurons and glial cells to produce IL-33, which then activates mast cells. IL-33 is related to initial neuroprotective immunity against CTE [106] (Figure 3E). HD is another uncommon autosomal dominant neurodegenerative disorder that is usually diagnosed in middle age and presents with cognitive, psychiatric, and motor symptoms. Its clinical manifestations are complicated and variable, with the disease deteriorating gradually. Most patients with HD die 15–20 years post-onset. Moreover, its onset can be insidious and characterized by slow progression. The major features of HD include dance-like movements, continuous cognitive and mental disorders, and dementia. Its etiology may be related to the expansion of glutamine repeats in the N-terminal region of Huntingtin (*HTT*), affecting diverse molecular pathways and ultimately causing neural impairment or degeneration [108]. One study in the literature demonstrated that the lack of an IL-1 receptor increased HTT protein accumulation in the striatum and exacerbated neurological symptoms and toxicity in HD mice [73]. Additionally, salivary IL-6 levels have been significantly associated with pathological symptoms and cognitive function in HD mutation carriers [74]. Several experiments have discovered that the inflammatory response in the brain and peripheral tissues is correlated with the pathological changes in HD. Currently, there is very little research on the physiological and pathological development process of IL-33 in HD, but future studies may elucidate IL-33′s critical effect on HD progression.

## 6. Clinical Trials on IL-33/ST2

The IL-33/ST2 pathway has a crucial effect on neurodegeneration; however, relevant clinical trials are limited. Nevertheless, research on the therapeutic application of IL-33 in other domains also exists. Itepekimab, a monoclonal antibody targeting the upstream signaling protein IL-33, was employed in phase 2 of a two-stage trial. It was administered subcutaneously to patients with moderate or severe asthma, followed by assessments of asthma control, lung function, and quality of life. The findings indicated that, in comparison to the control group, the degree of asthma control in patients decreased following itepekimab intervention. The underlying mechanism might involve IL-33 binding to its receptor (ST2) and the co-receptor IL-1 receptor accessory protein, initiating the downstream signaling pathway. This activation triggers cells of the innate and adaptive immune systems, resulting in type 2 and non-type 2 inflammation, potentially causing asthma and other respiratory diseases [94,109]. Allergens, air pollutants, and respiratory viruses encountered in daily life can exacerbate asthma and promote the release of IL-33. Clinical trials have demonstrated that the human IgG2 monoclonal antibody Astegolimab selectively inhibits the ST2 receptor of IL-33, which can reduce the asthma exacerbation rate (AER). Additionally, these trials have shown that Astegolimab is safe and well-tolerated [110]. The clinical application and mechanism of IL-33 in neurodegenerative diseases still require further investigation.

## 7. Discussion

The IL-33/ST2 pathway plays a crucial role in neurodegenerative diseases and is activated by tissue injury, fibrosis, remodeling, and inflammation; moreover, the pathway participates in the homeostasis or pathogenic mechanisms of such disorders. IL-33 acts not only as an alarmin but also as a cytokine involved in stimulatory signals. The precise IL-33 effect on cognition regulation in disease and healthy conditions is critical for regulating age-associated cognitive impairment and synaptic plasticity. Because of in-depth research, knowledge regarding IL-33 has expanded beyond its initial recognition as the factor inducing type-2 immunity. Currently, IL-33 is identified as a cytokine with multiple biological effects on congenital and acquired immunity. Several studies have explained IL-33’s functional and genetic roles in disease and healthy states. However, the in vivo expression of IL-33 in the bioactive form or its production in the disease state remains unclear. The bioactive forms of IL-33 in human disorders and the mechanisms underlying their entry into the corresponding target cells are unknown [26]. Moreover, research concerning IL-33′s regulatory components and the impact of single nucleotide polymorphisms (SNPs) in intron 1 and promoter on IL-33 expression and regulation is lacking [9]. Most studies have been performed in mouse models, and the extrapolation of these studies’ results to humans remains uncertain. The signaling pathways underlying the immunosuppressive and anti-inflammatory activity of IL-33 are only partially defined. The long-term safety and efficacy of IL-33 systemic administration in humans for neurodegenerative diseases such as AD remain unclear. The mechanisms underlying the transportation of IL-33 into the CNS after systemic administration is unknown. Therefore, future studies should focus on long-term efficacy and safety. These unknown problems are not only related to immunopathology but also related to regulatory mechanisms and genetic factors. Addressing these problems may pave the way for improvements in the utilization of current therapeutic tools and the development of new intervention strategies [56].

## Figures and Tables

**Table 1:** IL-33 distribution in human and mice tissues and cells.

Species	Conditions	Organs or Tissues	Cell Types	Refs.
human	Normal	brain	neuron, microglia, astrocyte, oligodendrocyte	[33,35]
		stomach	epithelium cell	[27]
		skin	keratinocytes	[27]
		lymph node and tonsil	endothelial cell, fibroblastic reticular cell	[27]
		colon, lung, cervix	endothelial cell	[28]
		blood vessels	endothelial cell	[28]
mouse	Normal	skin	keratinocytes	[27]
		lymph nodes, spleen, appendix	fibroblastic reticular cell	[28]
		adipose tissue	endothelial cell	[28]
		vagina, skin, lung, stomach, and salivary glands	epithelial cell	[27]
		eye (exception of optic nerve)	glial cells (the retinal inner nuclear layer)	[28]
		brain	neuron, microglia, astrocyte, oligodendrocyte	[34]
	Disease			
	LPS-induced endotoxin shock	liver	endothelial cell	[28]
	allergic airway inflammation	lung	epithelial cell	[28]
	DSS-induced colitis	colon	endothelial cell	[29]

**Table 2:** Role of IL-33 in neurodegenerative diseases.

Neurodegenerative Disease	Role of IL-33	Refs.
AD	IL-33 and ST2 are strongly expressed in the vicinity of Aps and NFTs and in glial cells in AD brains.	[58,59]
	Mice deficient in IL-33 showed Tau abnormalities and loss of neurons or synapses.	[60]
	IL-33 administration can promote the recruitment of microglia and increase the phagocytic activity of microglia to reduce Aβ pathological protein levels and amyloid plaque deposition.	[61]
	Following CBD treatment, the expression of IL-33 and TREM2 was increased, which ameliorated the symptoms of AD and decelerated the deterioration of cognitive abilities.	[62]
	The IL-33 gene may have a regulatory role in the development of cerebral amyloid angiopathy.	[63]
	IL-33 improves the pathological features of AD by promoting PU.1-dependent microglial state transition.	[64]
MS	The expression levels of IL-33 and ST2 are increased in both acute and chronic MS patients.	[65]
	The elevated levels of IL-33 in leukocytes of MS patients may activate the transcription factor NF-κB.	[66]
	IL-33 attenuates the development of EAE by converting the Th17/Th1 response to Th2 activity and via the polarization of anti-inflammatory M2 macrophages.	[67]
	Matrine by EAE rats IL-33/ST2 expression of the central nervous system.	[68]
PD	Serum levels of IL-33 and inflammatory factors are increased in PD patients.	[59]
	IL-33 enhances glial cell-mediated neuroinflammation.	[69]
ALS	MPO/HOCl promoted the apoptosis and iron death of motor neurons in an ALS mouse model, leading to neurological deficits.	[70]
	Prolonged IL-33 intervention delayed the onset of disease and attenuated astrocyte activation in mice.	[71]
	IL-33 levels are reduced in ALS patients, possibly because IL-33 is degraded by caspases.	[72]
CTE	Mast cell protease induces neurons and glial cells to produce IL-33 and then activates mast cells.	[48]
HD	The lack of an IL-1 receptor increased HTT protein accumulation in the striatum and exacerbated neurological symptoms and toxicity in HD mice.	[73]
	Salivary IL-6 levels were significantly associated with pathological symptoms and cognitive function in HD mutation carriers.	[74]
